# Supplementary material for: AffyMAPSDetector: a software tool to characterize Affymetrix GeneChip™ expression arrays with respect to SNPs
Source: BMC Bioinformatics. 2007 Jul 30;8:276. doi: 10.1186/1471-2105-8-276 (PMC1959249; doi:10.1186/1471-2105-8-276)
Supplement: Additional file 13 — AffyMAPSDetector v1 source code. AffyMAPSDetector source code. [file 1471-2105-8-276-S13.zip › AffyMAPSDetector_v1_src/doc/affychip/snpdetector/model/class-use/Probe.html]

Uses of Class affychip.snpdetector.model.Probe


|  |  |  |  |
| --- | --- | --- | --- |
| **Overview** \| **Package** \| **Class** \| **Use** \| **Tree** \| **Deprecated** \| **Index** \| **Help** | | |  |
| PREV   NEXT | **FRAMES**    **NO FRAMES**     **All Classes** |  |  |


---


## **Uses of Class affychip.snpdetector.model.Probe**

|  |  |
| --- | --- |
| Packages that use Probe | |
| **affychip.snpdetector.model** |  |

Uses of Probe in affychip.snpdetector.model

|  |  |
| --- | --- |
| Methods in affychip.snpdetector.model that return Probe | |
| `static Probe` | `SnpFinder.parseProbe(java.lang.String line)`             probe:HG-U95Av2:1000\_at:399:559; Interrogation\_Position=1367; Antisense;TCTCCTTTGCTGAGGCCTCCAGCTT |
| `Probe` | `ProbeSet.getProbeFor(java.lang.String x, java.lang.String y)` |

|  |  |
| --- | --- |
| Methods in affychip.snpdetector.model with parameters of type Probe | |
| `void` | `ProbeSet.addProbe(Probe aProbe)` |
| `void` | `GeneInfo.setProbeLocationIndicesFor(Probe aProbe)`             This method finds starting index, ending index and index of 13th position of a given probe sequence relative to this gene's mRNA sequence. |

---


|  |  |  |  |
| --- | --- | --- | --- |
| **Overview** \| **Package** \| **Class** \| **Use** \| **Tree** \| **Deprecated** \| **Index** \| **Help** | | |  |
| PREV   NEXT | **FRAMES**    **NO FRAMES**     **All Classes** |  |  |


---
